# Interstitial Lung Fibrosis Following COVID-19 Pneumonia

**DOI:** 10.3390/diagnostics12082028

**Published:** 2022-08-22

**Authors:** Mihai Lazar, Ecaterina Constanta Barbu, Cristina Emilia Chitu, Catalin Tiliscan, Laurentiu Stratan, Sorin Stefan Arama, Victoria Arama, Daniela Adriana Ion

**Affiliations:** 1 Faculty of Medicine, University of Medicine and Pharmacy Carol Davila, No. 37, Dionisie Lupu Street, Sector 2, 020021 Bucharest, Romania; 2 National Institute for Infectious Diseases Prof. Dr. Matei Bals, No. 1, Calistrat Grozovici Street, Sector 2, 021105 Bucharest, Romania

**Keywords:** pulmonary fibrosis, SARS-COV-2, long COVID, risk factors, fibrosis predictors, quantitative analysis

## Abstract

*Background and Objectives:* Pulmonary fibrosis represents a stage of normal physiologic response to inflammatory aggression, mostly self-limiting and reversible; however, numerous patients treated for SARS-CoV-2 pneumonia present after release from hospital residual lung fibrosis. In this article, we aim to present an optimization method for evaluating pulmonary fibrosis by quantitative analysis, to identify the risk factors/predictors for pulmonary fibrosis in patients with SARS-CoV-2 infection, and to characterize the impact of pulmonary fibrosis on the symptomatology of patients after release from the hospital. *Materials and Methods:* We performed a prospective observational study on 100 patients with severe forms of pneumonia, with a control group of 61 non-COVID normal patients. *Results:* We found persistent interstitial changes consistent with fibrotic changes in 69% of patients. The risk of fibrosis was proportional to the values of erythrocyte sedimentation rate (ESR), C reactive protein (CRP), and lactate dehydrogenase (LDH), and to the duration of hospitalization. The imaging parameters correlated with increased risk for interstitial fibrosis were the number of affected pulmonary lobes and the percent of interstitial pulmonary fibrosis. *Conclusions:* The main risk factors for pulmonary fibrosis post-COVID-19 identified in our study are increased ESR, CRP, LDH, duration of hospitalization and the severity of pneumonia.

## 1. Introduction

The first case of COVID-19 was first reported in Wuhan City, China, in December 2019 [1], causing an ongoing global pandemic. Severe acute respiratory syndrome coronavirus 2 (SARS-CoV-2) usually infects respiratory tract cells [2], but the extended viral tropism may induce a multisystemic disease [3] with increased death risk for the patient in the severe forms of this pathology. According to Aslan et al. [4], approximately one-third of hospitalized patients and 75% of COVID-19 patients admitted to the ICU develop acute respiratory distress syndrome (ARDS), with COVID-19 potentially being a leading cause of mortality in 2020 and 2021, with an estimate of 18.2 million deaths globally [5]. The severity of the disease is not limited to the acute episode; patients may develop late complications (neurologic, cardiac, digestive, and respiratory) included in the “long-COVID syndrome” or “post-acute COVID-19 syndrome” (PACS) [6]. The term PACS refers to persisting symptoms or abnormalities for at least four weeks from the moment of SARS-CoV2 infection [7]. After recovering from the acute episode of COVID-19 pneumonia, various new studies estimate that nearly 70–80% of the patients will continue to present post-infectious complications, especially in severe COVID-19 cases, with pulmonary fibrosis frequently reported as one of the more severe COVID-19 sequelae [8]. The most common lung problem faced by post-COVID patients is considered to be lung fibrosis, representing a real concern due to a large number of infections during the present pandemic [9]. The published studies communicated various prevalence rates of post-COVID-19 fibrosis ranging from 9.3% [10] to 84.15% [11]. In the evolution of pneumonia, the model of lesion repair includes three phases: injury, inflammation, and repair. In most pulmonary fibrotic conditions, dysregulation at one or more of these phases has been reported [12]. The persistence of a chronic inflammatory process of the lung has long been considered the main mechanism for interstitial lung fibrosis [13]. Computerized tomography (CT) evaluation represents the main tool for diagnosing interstitial lung fibrosis according to ATS/ERS/JRS/ALAT statement [14]. Pulmonary CT findings associated with pulmonary interstitial fibrosis include ground glass opacities, reticular opacities, traction bronchiectasis and bronchiolectasis, and honeycomb cysts [15]. Ground-glass opacity (GGO) represents an increased attenuation of the lung with preservation of bronchial and vascular margins; it is not to be confused with consolidation in which bronchovascular structures are obscured. It correlates with several pathogenic processes, such as partial filling of air spaces, interstitial inflammation, increased capillary blood volume, or fibrotic interstitial thickening [16] that are difficult to detect on thin-section CT. The presence of ground-glass opacities frequently represents a reversible disease process if it is not associated with other evidence of fibrosis, such as traction bronchiectasis [15]. The reticular pattern consists of a fine network of overlapping linear lines, indicating interstitial fibrosis. It is frequently associated with other fibrotic changes, such as architectural distortion and bronchiectasis. The honeycomb pattern consists of clustered cysts with well-defined walls, frequently in multiple layers, with subpleural topography, typically of comparable diameters (3–10 mm) but occasionally as large as 2.5 cm. The honeycomb pattern associates interstitial reticular changes, traction bronchiectasis, and GGO. Traction bronchiectasis and bronchiolectasis refer to dilatation of an airway adjacent to interstitial lung changes suggestive of fibrosis (GGO, reticular opacities, or honeycombing) [16,17].

In this article, we aim to present an optimization method in the evaluation of pulmonary fibrosis following COVID-19 pneumonia by quantitative analysis, to identify the risk factors/predictors for pulmonary fibrosis in patients with SARS-CoV-2 infection and to characterize the impact of pulmonary fibrosis on the symptomatology of patients with COVID-19 after release from the hospital.

## 2. Materials and Methods

In our observational and prospective study, we enrolled 100 patients with severe forms of COVID19 pneumonia who were admitted to our department between September and November 2021. The control group included additional 61 healthy subjects selected to match the age and sex ratio of the study group. We used the data from the control group to adjust the lung density measurement for an accurate estimate of lung fibrosis and as a reference for the radiological and biological parameters in the study group.

A severe form of COVID-19 was considered when at least one of the following criteria was present: peripheral oxygen saturation (SpO_2_) ≤93% in the ambient air, respiratory rate (RR) >30/min, arterial oxygen partial pressure to fractional inspired oxygen ratio (PaO_2_/FiO_2_ ratio) <300 or lung infiltrates >50% of lung parenchyma [18].

The following exclusion criteria were applied to all study participants: age under 18, pregnancy, prior known chronic pulmonary diseases: chronic obstructive pulmonary disease, chronic bronchitis, pulmonary emphysema, pulmonary tuberculosis, bronchiectasis, idiopathic interstitial lung disease, rheumatic diseases (rheumatoid arthritis, Sjögren syndrome, systemic lupus erythematosus, systemic sclerosis, etc.), smoking, toxic environmental exposures, chronic treatment with methotrexate, amiodarone or the presence of severe gastro-esophageal reflux documented before admittance.

### 2.1. Demographic and Biological Parameters

The age, sex, the duration of hospitalization/hospital stay for COVID-19, inflammatory parameters (fibrinogen, C reactive protein (CRP), erythrocyte sedimentation rate (ESR), serum ferritin), biochemical markers (lactate dehydrogenase, alanine transaminase, creatin-kinase), complete blood count (erythrocytes, leukocytes, lymphocytes, neutrophils), D-dimer, interleukin 1 (IL-1), and interleukin 6 (IL-6) were registered for every patient.

### 2.2. Imaging Parameters

All patients were evaluated by computer tomography (CT) scans with a 64 slices Definition AS (Siemens Healthcare GmbH, Munich, Germany) at hospital admission and 3 months after their release from the hospital. The acquisition was performed in helical mode with CAREDose4D, and CARE kV was activated to reduce the radiation dose, pitch 0.35, rotation time 0.5 s, collimation 1.2 mm, slice thickness 3 mm with an h additional reconstruction of 1.5 mm with H31f image filter for mediastinal window and H60f for the lung window. All patients were examined in inspiratory breath-hold and supine positions.

For evaluation of CT images, we used following density range scale: higher than 0UH—alveolar lesions (consolidations), between 0 and −200HU—mixed lesions (alveolar and interstitial), −201 to −800 HU—interstitial lesions, −801 to −1000 HU—normal lung parenchyma [19].

The evaluation software *syngoPulmo3D* was used for the quantitative analysis of lung lesions by measuring the percent of lung volume for each lesion category according to the same density range scale.

The imaging evaluation was blinded; the radiologist was unaware of the identity of the patients in the study group in both CT scans (performed at admission date and three months follow-up) and was also not informed if the patients were enrolled in the study group or the control group.

### 2.3. The Optimization Method in the Evaluation of Pulmonary Fibrosis Following COVID-19 Pneumonia by Quantitative Analysis

The quantitative analysis of lung fibrosis was performed on the three months follow-up CT scan. The patients were considered to have fibrosis if a specific pattern was observed on CT images performed at three months follow-up evaluations: ground glass, linear, reticular, honeycomb, and bronchiectasis.

Pulmonary fibrosis was quantified using syngoPulmo3D by measuring the percent of lung volume with densities between −201 and −800 HU for every patient on the three-month follow-up CT scan.

The density range −201 to −800 HU includes the fibrotic changes and small vessels that are normally found in every patient. The small vessels inside lung parenchyma have densities higher than −800 HU, which may increase overall lung density measured by software and lead to overestimation of fibrosis.

We quantified in the control group the percent of densities between −201 and −800 HU in normal lungs, corresponding to the percent of lung volume represented by the small vessels. The median value of this percent of lung volume was considered the correction factor for the measured lung fibrosis. For each patient in the study group (at 3 months follow-ups), we subtracted the correction factor from the measured percent of lung volume with fibrosis, obtaining the corrected lung fibrosis.

Lung fibrosis was characterized quantitatively by calculating the percent of lung volume with fibrosis and morphologically by presenting the fibrotic pattern distribution in the study group.

### 2.4. Identification of the Risk Factors/Predictors for Pulmonary Fibrosis in Patients with SARS-CoV-2 Infection

The relationship between the fibrosis and the registered patient data at admission was calculated using Spearman correlation, and the odds ratio (OR) was calculated by logistic regression.

Receiver operating characteristics (ROC) curves analysis was performed for all explanatory variables with an individual prediction of lung fibrosis to present their comparative performance. The optimal cutoff point was established by finding the most distant point on the ROC curve from the diagonal (Youden’s J).

Logistic regression analysis was performed to further characterize the relationship between fibrosis and various groups of parameters and obtain a fibrosis prognostic model. Correlations between the registered variables were assessed to avoid collinearity (R^2^ > 0.7; *p* < 0.001, VIF > 3). If any such pairs were found, the predictor variable with the lowest *p-*value was chosen to be included in the final multivariable analysis, and the other was ignored.

For the multivariable logistic regression, we performed a backward elimination method; the independent variables were excluded based on the results of the Wald test for the individual parameters obtained during logistic regression analysis; the least significant effect with *p* > 0.2 was removed from the prognostic model. The statistical significance of the regression model was estimated by the omnibus test of model coefficients. A *p*-value lower than 0.05 was considered statistically significant.

### 2.5. Characterization of the Impact of Pulmonary Fibrosis on the Symptomatology of Patients with COVID-19 after Release from the Hospital

The symptoms registered at three-month follow-up evaluations (fatigue, shortness of breath, chest pain, cough, memory loss, concentration problems, insomnia, palpitations, and dizziness) were assessed by anamnesis. Patient data are presented in numbers and percentages. The relationship between the fibrosis and the patient’s symptoms were calculated using Spearman correlation; statistical significance was evaluated by the Pearson Chi-Square test.

We used Statistical Package for Social Sciences (SPSS version 25, IBM Corp., Armonk, NY, USA) for the statistical analysis. Patient data are presented as medians and quartiles (Q1, Q3) for continuous variables and as percentages for the categorical variables. For the comparative evaluation of the three groups (patients at admission date, follow-up, and control group, respectively), we performed ANOVA with a post hoc LSD test. Spearman correlation, odds ratio (OR), and receiver operating characteristics (ROC) curves analysis were performed to identify the parameters associated with lung fibrosis. Multivariable logistic regression was performed to obtain a fibrosis prognostic model. A *p*-value lower than 0.05 was considered statistically significant.

## 3. Results

In our study 55% were male with median age 51 [43, 56] and 45% female with median age 58 [48, 66.5].

We observed a significant reduction in inflammatory parameters (CRP, ESR, fibrinogen, and ferritin), cytolytic markers (LDH, alanine transaminase), and neutrophils at follow-up compared to the admission date; we found no significant variation between the evaluation at three months and the control group (Table 1).

The duration of hospitalization was 17 days [15, 20]. The patients received during hospitalization corticoids for a median time of 11 days [9, 14] and antiviral treatment for 5 days [3, 6]; all patients received corticoids, 20% of patients received Tocilizumab, and 21% of patients received Anakinra.

The patients at admission presented with a median lung involvement of 36% of the total lung volume, consisting mainly of interstitial lesions (32%). The follow-up evaluations show an important regression of the interstitial lesions (17.3%) and a volume of normal lung densities of 73%, slightly lower than in the control group (77%) (Table 2).

### 3.1. Quantitative Analysis of Pulmonary Fibrosis (Changes Registered at Three Months Follow-Up)

**Figure 1:**
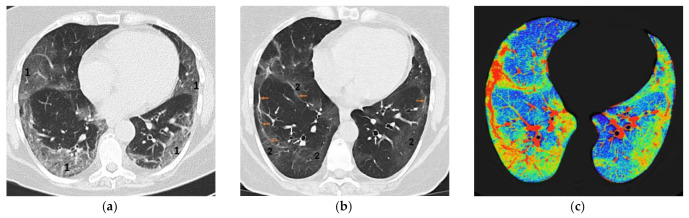
(**a**) Bilateral pneumonia with preponderant peripheric topography of inflammatory infiltrates (**1**); (**b**) bilateral fibrosis with linear (**orange arrows**) and “ground glass” pattern (**2**) and bronchiectasis images (**white arrows**); (**c**) bilateral fibrosis with color encoding (green—“ground glass fibrosis and thin reticular fibrosis; orange—linear fibrosis, blue—normal lung densities).

Thirty-one percent of patients presented no fibrosis, 34% presented mild fibrosis (1–9% of total lung volume), 19% had moderate fibrosis (10–19% of total lung volume), and 16% of patients were found with severe fibrosis (20–32% of total lung volume). The fibrosis patterns observed consisted of “linear” fibrosis in 64%, “ground glass” interstitial changes in 62% (Figure 1), “reticular” aspect in 15%, and “honeycomb” image in 4% of the patients. In mild fibrosis, the observed lesions were distributed subpleural, while in moderate and severe fibrosis, we observed the extension of fibrosis also in the central lung areas. We found traction bronchiectasis in 42% of patients, distributed mainly in the inferior lobes (39% of the patients), right medium lobe (21% of the patients), and superior lobes (17% of the patients).

While linear, thick reticular, and honeycomb patterns are identified and diagnosed easily, the mild “ground glass” and thin reticular patterns may be difficult to estimate accurately on the standard images. In this situation, the utilization of dedicated software to quantify the amount of pulmonary fibrosis is highly useful. The color encoding of densitometric ranges provides a lung fibrosis map that shows the extent of interstitial fibrosis (Figure 1), which is useful in monitoring and estimating pulmonary fibrosis.

### 3.2. Risk Factors/Predictors for Pulmonary Fibrosis

We evaluated further the correlations between the severity of fibrosis and the parameters registered as potential independent risk factors, and we calculated the OR for each parameter. The risk of fibrosis was found to be proportional to the values of ESR, CRP, lactate dehydrogenase (LDH), and the duration of hospitalization. For a 1 unit increase in the specified parameters, the risk of fibrosis increased by 5.7%, 1.5%, 0.5%, and 6%, respectively. We registered the highest correlations in the case of imaging parameters; for a 1 unit increase in the number of affected pulmonary lobes, the percent of interstitial pulmonary lesions, total pulmonary lung lesions, and the risk of fibrosis increases by 82%, 12.2%, and 8.1%, respectively. (Table 3)

**Figure 2:**
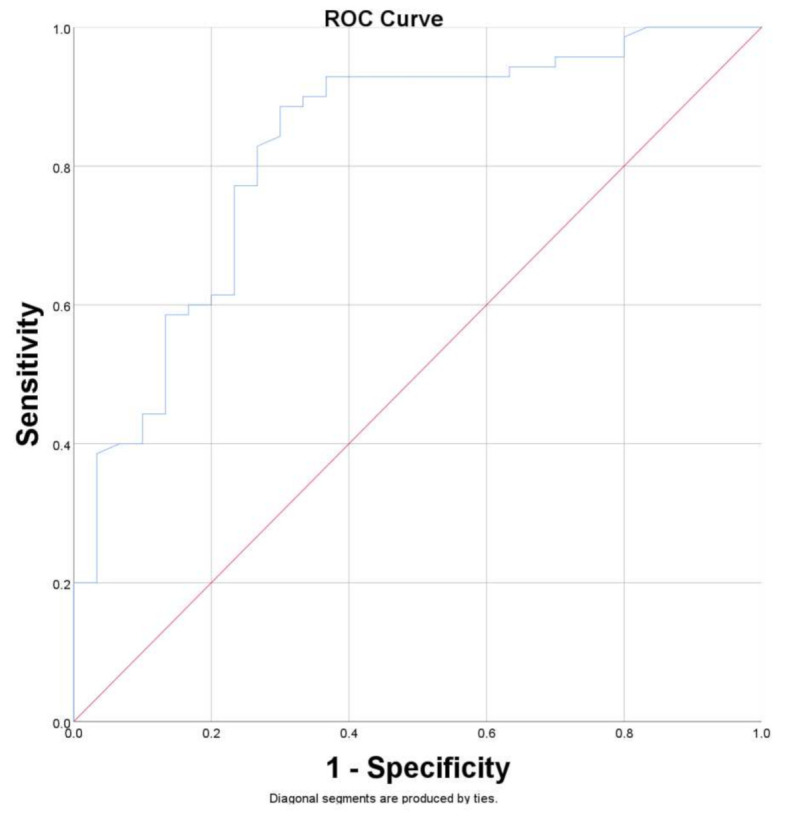
Receiver operating characteristics (ROC) curve for the ability of the “percent of interstitial pulmonary lesions” to predict pulmonary fibrosis. The area under curve (AUC) = 0.827 (0.736–0.918 CI 95%).

We performed ROC curves for all explanatory variables with the individual prediction of lung fibrosis to present their comparative performance (Table 4); “the percent of interstitial pulmonary lesions” presented the highest AUC value (Figure 2).

For a patient with a percent of 26.94% of interstitial lung involvement at admission to the hospital, we can predict the occurrence of late fibrosis with sensitivity (Se) of 0.83 and specificity (Sp) of 0.73, a higher percentage of initial lung involvement is associated with higher specificities for late lung fibrosis, with an Sp = 1 for 49% of interstitial lung involvement at admission.

We performed a further multivariable logistic regression analysis using the variables characterized in Table 3 and Table 4; the multivariable logistic regression model is presented in Table 5. The statistical significance of the multivariable logistic regression estimated by the omnibus test of model coefficients was lower than 0.001, with an overall accuracy prediction of 87.1%.

Based on the data in Table 5, we can also calculate the probability of interstitial fibrosis at three months using the following formula:

EXP (Constant + 0.034 × ESR + 0.082 × Duration of hospitalization + 0.36 × Duration of antiviral treatment − 2.524 × Percent of alveolar consolidation + 0.391 × Percent of interstitial pulmonary lesions)/[1 + EXP (Constant + 0.034 × ESR + 0.082 × Duration of hospitalization + 0.36 × Duration of antiviral treatment − 2.524 × Percent of alveolar consolidation + 0.391 × Percent of interstitial pulmonary lesions)].

### 3.3. Pulmonary Fibrosis and Symptomatology

Forty-three patients presented one or more persistent symptoms, not registered before an infectious episode, after three months of release from the hospital (Table 6). We found correlations with statistical significance between the presence of pulmonary fibrosis and fatigue, shortness of breath, cough, memory loss and concentration problems, and dizziness.

## 4. Discussion

### 4.1. Mechanisms of Post-COVID-19 Lung Fibrosis

The molecular mechanism leading to pulmonary fibrosis is still unclear, but it is considered to have a multifactorial basis. Pulmonary fibrosis has many inductors, starting from the endothelial lesions initiated by the viral aggression, followed by the release of fibrosis-inducing factors such as IL-6, transforming growth factor β (TGF-β), plasminogen activator inhibitor-1 (PAI-1) [20]. The intensity and duration of the inflammatory lung involvement play an important role in the persistent inflammatory processes. The M1 population of macrophages is substituted by M2 macrophages, which release pro-fibrotic mediators such as TGF-β and platelet-derived growth factor (PDGF), which activate the fibroblastic activation and proliferation [21]. The inflammatory process may induce a transition of alveolar epithelial cells into fibroblasts with fibrotic changes in both vitro and in vivo [22,23]. The high tropism of the SARS-CoV-2 viral spike protein for the angiotensin-converting enzyme-2 (ACE-2) receptor leads to downregulation of the level of the ACE2 receptor [24]. ACE-2 is considered to have a protective role in lung fibrosis. The decreased ACE-2 expression leads to high angiotensin 2 (ANG II) levels. ANG II is a potent vasoconstrictive peptide directly involved in the development of inflammation and fibrosis by signaling molecular events such as (a) production of pro-inflammatory cytokines (IL-6 and IL-8), (b) production of reactive oxygen species in infected alveolar cells, and (c) activation of TGF-β 1, leading to proliferation, migration, and differentiation of fibroblasts to myofibroblasts with resultant deposition of collagen and fibronectin [25].

COVID-19 patients are also considered to have an increased risk for pulmonary embolism (PE). Suh et al. [26] communicate an overall incidence rate of 16.5%, generating a supplementary decompensation of an already decreased lung function. The hypercoagulability state of COVID-19 patients predisposes them to develop chronic PE, which could also exacerbate the progression of PF [27].

### 4.2. Evaluation of Pulmonary Fibrosis Following COVID-19 Pneumonia

According to Liu et al. [28], the patients may develop pulmonary fibrosis right after discharge from the hospital or several weeks later. Zou et al. [11] demonstrated a diminishing of pulmonary fibrosis during follow-up in some patients, with its persistence in most cases. Diminishing of fibrosis was also confirmed by Nabahati et al. [29] in one-third of the patients when comparing the 3 months and 6 months post-COVID-19 evaluations, while for approximately two-thirds of the patients, no important changes were found.

The dynamics of the long-lasting sequels in patients who have recovered from severe COVID indicate that there is a 30% chance of developing persistent respiratory system pathology and a 10% chance of developing a severe pathology; this includes the development of persistent fibrotic lung damage (38%) during the first 12 months after diagnosis in COVID patients [30]. Other authors described persistent fibrosis of 52% at 3 months follow-up, with persistent similar radiologic changes at 6-month follow-up in 66.1% and diminished in 33.9% of cases [29]. Zhao et al. found fibrotic changes in 70.91% of patients evaluated 3 months after discharge with lung abnormalities on spirometry in 25.45% [31]. In our study, performed on patients with severe forms of disease and extensive inflammatory lung involvement, we found 69% residual fibrosis 3 months after the release from the hospital, with associated symptoms in 43% of patients.

Ground-glass opacities, consolidations, and even reticular changes may be found in the acute phase of pneumonia and can persist after the resolution of the symptomatology [9]. We observed that our patients presented ground-glass opacities at distance from the acute episode (three months), with inflammatory and cytolytic markers in normal ranges, and all of them tested negative for SARS-CoV-2 at three months. The ground glass changes were associated with reticular changes, honeycomb images, and traction bronchiectasis. In this context, we interpreted the residual ground glass changes as interstitial fibrosis.

The classic diagnosis of interstitial pulmonary fibrosis includes a reticular or reticulonodular pattern associated with decreased lung volumes and, in later stages, cystic areas representing honeycomb lung [32]. When a positive diagnosis of interstitial pulmonary fibrosis is performed on the chest radiograph, the real diagnosis is obtained in 48 to 87% of cases [33,34]. CT characteristics of interstitial pulmonary fibrosis include patchy, predominantly peripheral, subpleural, and symmetrical basal honeycombing, reticular abnormalities, and “ground glass” opacities [35,36]. Honeycombing, distorted lung architecture, and traction bronchiectasis represent reliable radiological indicators to start empiric antifibrotic drugs, with the highest concurrence of the expert groups for honeycombing [19]. In the present study, we recommend an additional marker, “percent of interstitial pulmonary lesions,” to identify the patients who will develop fibrosis; for 26.94% of interstitial lung involvement at hospital admission, we anticipate the occurrence of pulmonary fibrosis with Se = 0.83 and Sp = 0.73. We intend to perform follow-ups after one year and two years of release from the hospital to evaluate the grade of long-term fibrosis.

Fibrose severity can be quantified using visual assessment or by objective semi-automated post-processing of CT data. The visual estimation of fibrosis can be reported as mild, moderate, or severe, or can be presented as an estimate of the percentage of lung affected to the nearest 5%, 10%, or 25% is highly operator dependent. A hybrid method is to divide the lungs into upper, mid, and lower zones; this approach can be applied to both interspaced and volumetric CT datasets [37]. Although many studies with trained observers showed high levels of reproducibility, observer variation is a problematic aspect of visual CT assessment; quantitative image analysis of lung fibrosis is rapidly changing and can prove useful in assessing the extent of fibrosis [37].

Several studies have shown that a positive diagnosis of interstitial lung fibrosis can be met on a CT with a specificity of over 95% if typical findings are present [35,38,39]. Although a CT examination with characteristic findings is highly specific for interstitial lung fibrosis, only 37% to 67% of patients with histological interstitial lung fibrosis can be identified using a CT scan [35,38,40]. Therefore, we may consider that CT examinations underestimate fibrosis in post-COVID-19 patients, and in the case of persistent symptomatology, interstitial lung fibrosis should not be excluded only based on CT evaluation.

### 4.3. Risk Factors/Predictors for Pulmonary Fibrosis in Patients with SARS-CoV-2 Infection

A higher intensity of the inflammatory process, associated with higher levels of CRP and IL-6 in patients [41], might lead to lung fibrosis during recovery. In our study performed on patients with severe forms of pneumonia, we found that ESR has a higher correlation with fibrosis than CRP (Spearman’s rho 0.422 vs. 0.252) with a higher risk of fibrosis (5.7% vs. 1.5% for 1 unit increase in the variable). IL-6 presented a significant variation between the value registered at admission and three months follow-ups; however, no significant correlation with the occurrence of lung fibrosis has been found.

Huang et al. communicated that increased levels of neutrophils, Ne/Ly ratio (NLR), CRP, and LDH during hospitalization were associated with extensive fibrosis at the two-month follow-up [42]. In our study, we found differences with statistical significance in neutrophils and LDH values between the initial evaluation and three-month follow-up, with only LDH showing a significant individual association with lung fibrosis.

A high disease severity demonstrated by CT scan and a longer duration of illness has been documented to have a significant impact on post covid lung fibrosis [43]. The more severe forms of the disease, the longer durations of hospitalization were, and they were more frequently associated with pulmonary fibrosis. The patients with post-COVID-19 lung fibrosis presented a mean of 21.58 days of hospitalization, compared with 12.65 days in non-fibrotic patients [8]. Administration of anti-IL-6, Tocilizumab, Sarilumab, and glucocorticosteroids could improve the evolution of critically ill patients with COVID-19 [44] but are unable to prevent the occurrence of pulmonary fibrosis.

Proper timing of initiation of BIPAP/NIV therapy and its early use in comorbid class was associated with a lower percent of lung fibrosis; however, prolonged exposure to high concentrations of oxygen increases the production of oxygen-derived free radicals with increased damage to the alveolar epithelium [43,45].

In cases of severe COVID-19 patients, the length of stay in an intensive care unit, the use of high flow nasal oxygen, the need for mechanical ventilation, and the presence of ARDS have also been associated with a higher risk of occurrence of pulmonary fibrosis [46,47]. The risk of lung fibrosis development is considered to be higher for the elderly patient, necessitating ventilatory support [9].

Half of the patients (52%) with moderate and severe disease present coagulation abnormalities linked with the presence of inflammation and older age; therefore, targeting the mechanisms underlying coagulopathy and inflammation may constitute new important therapeutic strategies for the treatment of this complex pathology [48].

In our study, CRP, ESR, LDH, duration of hospitalization, duration of antiviral treatment, the number of affected pulmonary lobes, the percent of alveolar consolidation, the percent of mixed pulmonary lesions, the percent of interstitial pulmonary lesions at hospitalization were significantly independently associated with the presence of fibrosis during follow-up. The highest association with pulmonary fibrosis was presented by the percent of interstitial pulmonary lesions at admission. The percent of alveolar consolidation and interstitial pulmonary lesions was the only variable independently associated with the presence of fibrosis in multivariate analysis. Our finds were consistent with the observation of Han et al., who communicated that a more severe initial CT lung involvement was independently associated with permanent lung fibrosis [49]. The percent of interstitial pulmonary lesions presented a higher correlation and higher OR with pulmonary fibrosis than alveolar consolidation lesions; therefore, evaluating the percent of interstitial inflammatory changes represents a better option if the fibrosis risk needs to be estimated based on a single predicting factor.

### 4.4. Impact of Pulmonary Fibrosis on the Symptomatology of Patients with COVID-19 after Release from the Hospital

In a systematic review of 618 articles, Hama et al. [8] communicated that post-COVID-19 fibrosis had a prevalence of 44.9%, with the most common and persistent symptoms of dyspnea (50%), cough (31.6%), chest pain (30.5%), fatigue (80%), and myalgia (58.3%) (*p*-value < 0.05), consistent with our findings. In our study, the patients presented at three months follow-up fatigue, shortness of breath, chest pain, cough, memory loss, concentration problems, insomnia, palpitations, and dizziness had significant association with lung fibrosis.

Aprospective study performed by Arnold et al. on 131 participants [50] demonstrates the persistence of symptoms at 8–12 weeks in most patients, even those admitted with mild disease; most patients (74%) presented breathlessness and excessive fatigue. Huang et al. [51] found at 6 months after release from hospital that 76% of patients (1265 of 1655) presented at least one symptom; fatigue was the most common symptom present in 63% of cases, followed by sleep difficulties (26%), hair loss in 22% cases, and smell disorder (11%). Isolated cases of diabetes and thyroiditis have been reported after release from the hospital [52,53]; in severe cases with prolonged immobilization and steroid administration, bone demineralization may occur, accentuated by eventual vitamin D deficiency developed during or after COVID-19 [54].

After release from the hospital, psychological aspects also need to be addressed. In a study performed at 38 hospitals, Chopra et al. [55] communicated that nearly half of all patients (238 of 488) reported being emotionally affected by their health, and 28 sought care for mental health.

Given the diversity of symptoms, the management of these patients cannot be limited to a single specialized clinic and requires a multidisciplinary team approach and a set of investigations personalized for each patient, considering the rationalization of healthcare resources, especially in a timeframe when the health services are overloaded with COVID-19 patients [56].

### 4.5. Treatment of Post-COVID-19 Pulmonary Fibrosis

Currently, there are various treatment strategies under evaluation. Prolonged use of anti-viral drugs, anti-inflammatory, and anti-fibrotic medication to reduce the probability of development of lung fibrosis has been proposed [56].

The prolonged treatment with corticosteroids in preventing post-COVID-19 pulmonary fibrosis may be useful in subgroups of patients, such as those with tomographic abnormalities suggestive of organizing pneumonia [57]. Some authors report a higher prevalence of lung fibrosis by three folds in patients who received steroid treatment [8]; however, prolonged steroid treatment is usually administrated in severe forms of pneumonia, and lung fibrosis may be associated with the severity of pneumonia and not with the corticoid treatment. Corticoid treatment did not demonstrate important benefits to critical COVID-19 patients [58]; however, corticosteroid therapy in patients with ARDS was shown to improve the inflammatory storm and reduce the length of disease [59] and the risk of lung fibrosis.

Administration of anti-fibrotic drugs, such as nintedanib and pirfenidone, can be a solution even from the acute phase of pneumonia because they also have an anti-inflammatory effect [60]. Pirfenidone ameliorates lipopolysaccharide-induced pulmonary inflammation and fibrosis by blocking NLRP3 inflammasome activation and reducing lung injury induced by COVID-19 pneumonia [61]; it also reduces plasmatic and pulmonary IL-6 concentration. Although IL-6 has a profibrotic effect, an experimental study suggested that inhibition of IL-6 in the early phase of lung injury induces fibrosis, while inhibition of IL-6 in later stages of pneumonia and at beginning of the fibrotic phase might ameliorate the lung fibrosis [62].

These antifibrotic drugs should be considered for patients with a progressive decline of respiratory function during follow-up, although randomized controlled trials are needed to respond to this hypothesis [57].

Diethylcarbamazine, an ant filarial agent, was also communicated as a possible treatment variant due to its immunomodulatory, anti-inflammatory, and antifibrotic effects [63].

There are many pathologies associated with the risk of developing interstitial pulmonary fibrosis, such as smoking, toxic environmental exposures, diabetes mellitus, gastroesophageal reflux disease, genetic factors, chronic obstructive pulmonary disease, chronic bronchitis, pulmonary emphysema, pulmonary tuberculosis, bronchiectasis, idiopathic interstitial lung disease or associated with rheumatic diseases (rheumatoid arthritis, Sjogren syndrome, systemic lupus erythematosus, systemic sclerosis, etc.) [64,65,66], which should be taken into consideration when evaluating interstitial lung fibrosis in post-COVID-19 patients. The interstitial fibrotic changes induced by COVID-19 may be overestimated in the situation of a patient who presents another pathology that can determine interstitial fibrosis; therefore, in our study, all the above-mentioned pathologies represented exclusion criteria.

PACS represents a global problem that necessitates supplementary research and resources for a better understanding of the mechanisms, risk factors, and manifestations to establish the optimal management of the patients.

*Study limitations*: The diagnosis of interstitial lung fibrosis was established by CT scan only, no lung function tests were performed at follow-up to characterize the interstitial lung fibrosis.

## 5. Conclusions

The main risk factors for pulmonary fibrosis post-COVID-19 identified in our study are increased ESR, CRP, LDH, duration of hospitalization, and the severity of pneumonia (characterized by several pulmonary lobes involved and the percent of interstitial pulmonary lesions). “Percent of interstitial pulmonary lesions” can be used as a marker to identify the patients who will develop fibrosis, with Se = 0.83 and Sp = 0.73 for a percent of 26.94% of interstitial lung involvement. We found correlations with statistical significance between the presence of pulmonary fibrosis in patients post-COVID-19 and fatigue, shortness of breath, cough, memory loss and concentration problems, and dizziness.

## Figures and Tables

**Table 1:** Biologic parameters in study group and control group.

Parameter	Admission DateMedian [Q1, Q3]	Follow-Up (3 Months) Median [Q1, Q3]	Control Group Median [Q1, Q3]	*p*-Value Admission vs. Follow-Up	*p*-Value Follow-Up vs. Control
C reactive protein (mg/L)	43.9 [12.8, 89.9]	3.35 [1.85, 6,49]	2.8 [2.3, 3.6]	<0.001	0.794
Erythrocyte Sedimentation Ratio (mm/h)	45.8 [18.9, 60.7]	15 [12, 17]	14 [11, 16]	<0.001	0.570
Fibrinogen (mg/dL)	479 [368.8, 609.5]	344 [307, 413.5]	303.5 [244, 340.25]	<0.001	0.007
Lactate dehydrogenase (U/L)	279.2 [230.7, 365.1]	188 [168, 214]	180.5 [160, 197.75]	<0.001	0.419
Alanine transaminase (U/L)	42.2 [28, 57.6]	37.5 [32, 47.7]	36 [31.5, 39.5]	<0.001	0.151
Creatine kinase (U/L)	112.5 [48, 166.7]	71.5 [36.7, 182]	75 [41, 127]	0.222	0.144
Erythrocytes (×10^6^/µL)	4.68 [4.2, 5]	4.7 [4.3, 5.1]	4.64 [4.28, 5]	0.665	0.519
Leukocytes (×10^3^/µL)	6.6 [5.3, 10]	7.1 [5.8, 9.1]	6.6 [5.5, 9.1]	0.096	0.644
Lymphocytes (×10^3^/µL)	1 [0.6, 1.6]	1.5 [1.1, 2.1]	1.5 [1.3, 2]	0.746	0.998
Neutrophils (×10^3^/µL)	4.9 [3.4, 7.5]	4.1 [3.2, 4.5]	4.1 [3.2, 4.4]	<0.001	0.925
Ly/Ne ratio	0.2 [0,1, 0,4]	0.4 [0.3, 0.5]	0.4 [0.3, 0.5]	0.383	0.9
Platelets (×10^3^/µL)	203.3 [150.7, 256]	223 [173.5, 301.7]	225 [186, 280.5]	0.614	0.937
Ferritin (ng/mL)	667.1 [319.4,1236.6]	285 [150, 409.6]	238 [150, 373.6]	<0.001	0.603
D-dimers (ng/mL)	204 [148.5, 287.2]	210 [154, 283.5]	201 [153.2, 257]	0.111	0.658
IL-1 (pg/mL)	4.19 [0.2, 14.2]	3.7 [0.4, 9.8]	2.6 [0.3, 5.8]	0.058	0.763
IL-6 (pg/mL)	68.6 [34.3, 249.1]	8.7 [4.4, 24.2]	5.1 [3.3, 7]	<0.001	0.814

Q1-first quartile, Q3-third quartile.

**Table 2:** Radiologic parameters in the study groups.

Parameter	Admission Date	Follow-Up (3 Months)	Control Group	*p*-ValueAdmission vs. Follow-Up	*p*-Value Follow-Up vs. Control
Affected pulmonary lobes (*n*, median, Q1, Q3)	5 [4, 5]	2.7 [0, 5]	0	<0.001	<0.001
Consolidation (%, median, Q1, Q3)	1 [0.7, 1.7]	0.7 [0.5, 0.9]	0.6 [0.5, 0.7]	<0.001	0.252
Mixed lesions (%, median, Q1, Q3)	2.6 [1.4, 3.9]	1.2 [1, 1.5]	0.8 [0.7, 0.9]	<0.001	0.068
Interstitial lesions (%, median, Q1, Q3)	32.2 [23.3, 45.5]	17.3 [14, 27]	9 [7.7, 10.5]	<0.001	<0.001
Normal pulmonary densities (%, median, Q1, Q3)	58.6 [44.4, 67.9]	72.9 [66.4, 75]	77.4 [75.9, 80.9]	0.001	<0.001
Total pulmonary lesions (%, median, Q1, Q3)	36.3 [25.3, 53]	19.4 [15.7, 28.7]	10.3 [9, 12.05]	<0.001	<0.001

**Table 3:** Risk factors associated with pulmonary fibrosis in patients at hospital admission.

Parameter	Spearman’s Rho	*p*-Value	OR [CI]	Risk of Fibrosis
Age	130	0.198		
CRP	0.252	0.02	1.015 [1.002, 1.028]	1.5% *
ESR	0.422	<0.001	1.057 [1.021, 1.094]	5.7% *
Fibrinogen	0.192	0.06		
LDH	0.204	0.05	1.005 [1, 1.01]	0.5% *
Alanine transaminase	0.135	0.19		
Creatine kinase	−0.74	0.47		
Erythrocytes	0.007	0.94		
Leukocytes	0.122	0.234		
Lymphocytes	0.102	0.321		
Neutrophils	0.076	0.457		
Ly/Ne ratio	0.052	0.615		
Ferritin	−0.077	0.518		
Platelets	0.152	0.137		
D-dimers	0.12	0.25		
IL-1	−0.206	0.302		
IL-6	0.192	0.145		
Duration of hospitalization	0.249	0.012	1.06 [1.008, 1.115]	6% *
Duration of antiviral treatment	0.246	0.019	1.089 [0.957, 1.239]	−
Number of affected pulmonary lobes	0.252	0.012	1.82 [1.175, 2.818]	82% *
Percent of alveolar consolidation	0.389	<0.001	1.124 [0.776, 1.628]	−
Percent of mixed pulmonary lesions	0.357	<0.001	1.089 [0.917, 1.295]	−
Percent of interstitial pulmonary lesions	0.519	<0.001	1.122 [1.065, 1.183]	12.2% *
Percent of normal lung densities	−0.478	<0.001	0.924 [0.888, 0.761]	8.6% #
Total pulmonary lung lesions	0.480	<0.001	1.081 [1.04, 1.124]	8.1% *

* For a 1 unit increase in the parameter; # for 1 unit decrease in the parameter.

**Table 4:** ROC curve analysis for the variables with individual prediction of lung fibrosis.

Predictor	AUC	Std Error	*p*-Value	CI 95%	
				Lower Bound	Upper Bound
CRP	0.672	0.06	0.021	0.547	0.797
ESR	0.787	0.07	0.001	0.649	0.925
LDH	0.629	0.06	0.05	0.509	0.749
Duration of hospitalization	0.656	0.06	0.014	0.537	0.774
Duration of antiviral treatment	0.653	0.07	0.023	0.519	0.786
Number of affected pulmonary lobes	0.630	0.06	0.041	0.503	0.756
Percent of alveolar consolidation	0.745	0.06	<0.001	0.622	0.868
Percent of mixed pulmonary lesions	0.725	0.06	<0.001	0.597	0.853
Percent of interstitial pulmonary lesions	0.827	0.04	<0.001	0.736	0.918
Percent of normal lung densities	0.801	0.05	<0.001	0.691	0.911
Total pulmonary lung lesions	0.803	0.05	<0.001	0.695	0.910

**Table 5:** Multivariable logistic regression model for patients with lung fibrosis.

Variable	B	S.E.	Wald	*p*	OR	95% CI for OR	
						Lower	Upper
ESR	0.034	0.023	2.165	0.141	1.035	0.989	1.083
Duration of hospitalization	0.082	0.055	2.198	0.138	1.086	0.974	1.21
Duration of antiviral treatment	0.36	0.199	3.264	0.071	1.433	0.970	2.118
Percent of alveolar consolidation	−2.524	1.120	5.076	0.024	0.08	0.009	0.720
Percent of interstitial pulmonary lesions	0.391	0.176	4.913	0.027	1.478	1.046	2.089
Constant	−9.789	4.092	5.722	0.017	0		

**Table 6:** Symptoms for patients at 3 months and correlations with pulmonary fibrosis.

Parameter	Number of Patients (*n*,%)	Spearman’s Rho	*p*-Value
Fatigue	32 (32%)	0.449	<0.001
Shortness of breath	20 (20%)	0.327	0.001
Chest pain	11 (11%)	0.091	0.365
Cough	16 (16%)	0.286	0.004
Memory loss and concentration problems	11 (11%)	0.230	0.021
Insomnia	16 (16%)	0.048	0.634
Palpitations	11 (11%)	0.021	0.834
Dizziness	13 (13%)	0.253	0.011

## Data Availability

The data presented in this study are available on request from the corresponding author.
